# Molecular and Functional Analyses of the Fast Skeletal Myosin Light Chain2 Gene of the Korean Oily Bitterling, *Acheilognathus koreensis*

**DOI:** 10.3390/ijms140816672

**Published:** 2013-08-13

**Authors:** Hee Jeong Kong, Ye-Ji Lee, Woo-Jin Kim, Hyung Soo Kim, Bong-Seok Kim, Cheul Min An, Sang-Yeob Yeo, Hyun Kook Cho

**Affiliations:** 1 Biotechnology Research Division, National Fisheries Research and Development Institute, Busan 619-705, Korea; E-Mails: orange7654@naver.com (Y.-J.L.); wj2464@korea.kr (W.-J.K.); ecomarine@korea.kr (H.S.K.); bskim2002@korea.kr (B.-S.K.); ancm@korea.kr (C.M.A.); 2 Division of Applied Chemistry and Biotechnology, Hanbat National University, Daejeon 305-719, Korea; E-Mail: yeosy67@gmail.com; 3 Department of Molecular Biology, Pusan National University, Busan 609-735, Korea

**Keywords:** *Acheilognathus koreenis*, C/EBPβ, expression, Korean oily bitterling, myosin light chain 2, promoter assay

## Abstract

We identified and characterized the primary structure of the Korean oily bitterling *Acheilognathus koreensis* fast skeletal myosin light chain 2 (*Akmlc2f*), gene. Encoded by seven exons spanning 3955 bp, the deduced 168-amino acid AkMLC2f polypeptide contained an EF-hand calcium-binding motif and showed strong homology (80%–98%) with the MLC2 proteins of *Ictalurus punctatus* and other species, including mammals. *Akmlc2f* mRNA was highly enriched in skeletal muscles, and was detectable in other tissues. The upstream regions of *Akmlc2f* included a TATA box, one copy of a putative MEF-2 binding site and several putative C/EBPβ binding sites. The functional activity of the promoter region of *Akmlc2f* was examined using luciferase and red fluorescent protein reporters. The *Akmlc2f* promoter-driven reporter expressions were detected and increased by the C/EBPβ transcription factor in HEK293T cells. The activity of the promoter of *Akmlc2f* was also confirmed in the developing zebrafish embryo. Although the detailed mechanism underlying the expression of *Akmlc2f* remains unknown, these results suggest the muscle-specific expression of *Akmlc2f* transcript and the functional activation of *Akmlc2f* promoter by C/EBPβ.

## 1. Introduction

Myosins are ATP-dependent motor proteins that generate force by interacting with actin. They are well known for their role in muscle contraction and in a range of other eukaryotic motility processes including cytokinesis, karyokinesis and cell migration [1,2]. Myosins are classified into two classes: the conventional two-headed myosins and the unconventional single-headed myosins [3]. The two-headed myosins are hexameric proteins consisting of two myosin heavy chains (MHC; 200 kDa) and four myosin light chains (MLC; 20 kDa) (two essential and two regulatory light chains) [4]. Each MHC consists of a structural α-helical rod portion and a globular head. MLCs provide stabilization by wrapping around the α-helical neck region of the MHC in an anti-parallel orientation [1].

MLCs comprise two sub-families: the essential- or alkali-light chain (MLC-1 or ELC) that can be released from heavy chains by alkali treatment, and the regulatory light chain (MLC-2 or RLC) that is removable by 5,5′-dithio-bis-2-nitrobensoic acid (DTNB) treatment [5,6]. MLC-2 is phosphorylated by Ca^2+^/calmodulin-dependent myosin light chain kinase (MLCK) activated through the influx of Ca^2+^ in smooth muscle [7]. This phosphorylation enables the myosin crossbridge to bind to the actin filament, allowing contraction to begin. MLCK is important in the contraction mechanism in muscle; the most important regulator of MLCK activity is [Ca^2+^]i. In addition to MLCK, various kinase such as protein kinase C (PKC), Rho-kinase (ROCK), and myotonic dystrophy kinase-related Cdc42-binding kinase (MRCK) regulate the function of MLC2 by phosphorylation [8,9].

In fish, *mlc2* gene promoters were identified in zebrafish, rainbow trout, gilthead sea bream and marine medaka [10–14]; the activities of the *mlc2* promoters were demonstrated using the chloramphenicol acetyltransferase (CAT) reporter gene, LacZ reporter gene, green fluorescence protein (GFP) gene, and red fluorescence protein (RFP) gene. The promoters contained several putative myocyte-specific enhancer factor 2 (MEF-2) and E-box binding sites, which bind myogenic basic helix-loop-helix transcription factors that play key roles in muscle differentiation.

Korean oily bitterling *Acheilognathus koreensis* is a Korean endemic cyprinid found in freshwater [15]. It is distributed in the rivers flowing into the South Sea or the West Sea towards the south of Geumgang. The laterally flattened spindle-shaped body of the oily bitterling is dark-brown on the dorsal side and light-brown on the ventral side. As part of a recent species preservation effort, genetic studies of Korean oily bitterling have been initiated; Hwang *et al.* reported the complete mitochondrial genome sequence of *A. koreensis* [16]. Herein, we report the molecular cloning and characterization of the *mlc2f* gene of Korean oily bitterling (*Akmlc2f*) at the genomic DNA and mRNA levels. We analyzed pairwise and multiple alignments of the deduced AkMLC2f polypeptide sequence and other MLC2 homologs. No studies have reported the transcriptional regulation of fish *mlc2f* genes; therefore, we investigated the transcriptional regulation of the *Akmlc2f* gene using the luciferase or DsRed reporter assay system. Additionally, we investigated the tissue distribution of *Akmlc2f* transcript. This study is the first to report the molecular and functional analyses of the Korean oily bitterling *mlc2f* gene.

## 2. Results and Discussion

### 2.1. Characteristics of *Akmlc2f* cDNA, Genomic DNA and Deduced Amino Acid Sequences

**Figure 1:**
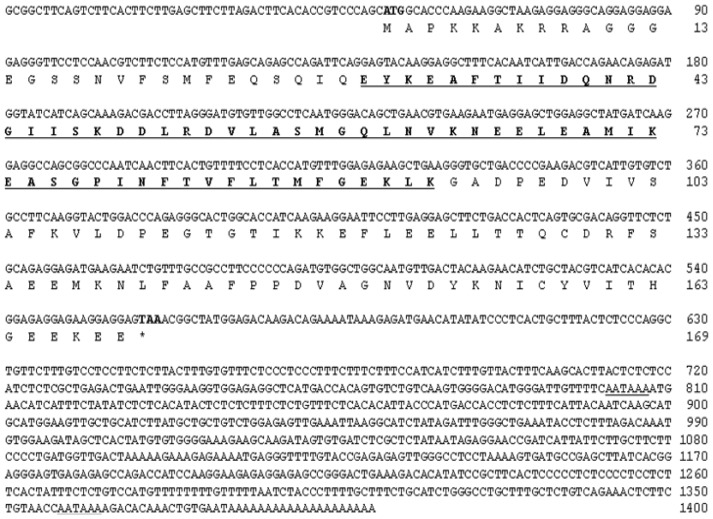
Nucleotide and deduced amino acid sequences of *Akmlc2f* cDNA. Start (ATG) and stop (TAA) codons are in bold. The EF-hand calcium-binding motif is in bold and underlined. The polyadenylation signals are underlined.

The expressed sequence tag (EST) clone, AK-1-2a-O18, contained a 1247-bp insertion that included the open reading frame (ORF) and 3′-untranslated region (UTR), and which showed significant sequence homology to known *mlc2f* sequences. The 5′-UTR of Korean oily bitterling *mlc2f* was amplified from the cDNA of muscle tissue using 5′-rapid amplification of cDNA ends (RACE). The full-length *Akmlc2f* cDNA sequence was 1400-nt in length and contained a 507-nt ORF encoding a 169-aa protein, preceded by a 51-nt 5′UTR, and followed by an 842-nt 3′UTR (GenBank accession no. KF192922, Figure 1).

A database search using the BLASTP software (http://www.ncbi.nlm.nih.gov/Blast.cgi, NCBI, Bethesda, MD, USA) revealed that the deduced amino acid sequence of AkMLC2f contained an EF-hand calcium-binding motif (Figure 1). A key difference between MLC2 and MLC1 is a serine residue in the *N*-terminus of MLC2, which is absent in MLC1 [17]. MLC2 is phosphorylated by Ca^2+^/calmodulin-dependent MLCK at the serine residue, allowing myosin ATPase activation by actin and muscle contraction [1]. The potential phosphorylation site at Ser17 of AkMLC2f was predicted using the GPS2.1.2. The theoretical isoelectric point (pI) and molecular weight (MW) of the deduced AkMLC2f were calculated using the ExPASy website, with a predicted pI and MW of 4.65 and 18.8 kDa, respectively.

**Figure 2:**
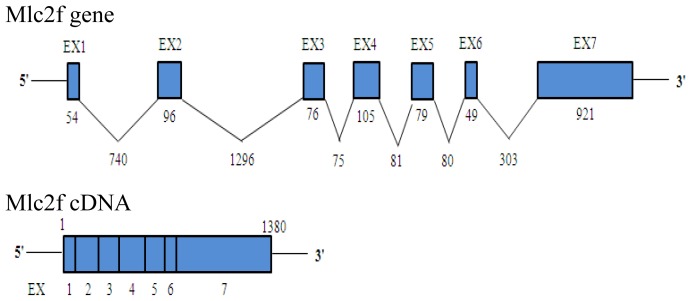
Schematic representation of the genomic organization of the *Akmlc2f* gene. The exon/intron structure is shown and the numbers indicate the length of exon/intron.

The *Akmlc2f* gene was isolated by screening a Korean oily bitterling fosmid library using primers based on the *Akmlc2f* cDNA sequence. Fosmid clone #17-H18 contained the *Akmlc2f* gene. The genomic organization of the *Akmlc2f* gene included seven exons interrupted by six introns (GenBank accession no. KF192924). Exons 1–7 were 54, 96, 76, 105, 79, 49, and 921 nt respectively; introns 1–6 were 740, 1296, 75, 81, 80, and 303 nt, respectively, with a conserved GT/AG splice site at each exon-intron junction (Figure 2). The seven-exon structure of the *Akmlc2f* gene is conserved in vertebrate orthologs [14]. The genomic region in the Javanese ricefish *mlc2f* gene contains duplicated and repetitive sequences in introns 1 and 3, respectively [14]; however, the intron regions of the *Akmlc2f* gene did not contain such long and complex duplicated or repetitive sequences. Instead, intron 1 of the *Akmlc2f* gene contained short duplicated sequences (TATAAAAAATGATATTAAATT, 21 bp) beginning 334 bp from the 5′-end of intron 1.

### 2.2. Pairwise Multiple Alignment of *Akmlc2f*

**Figure 3:**
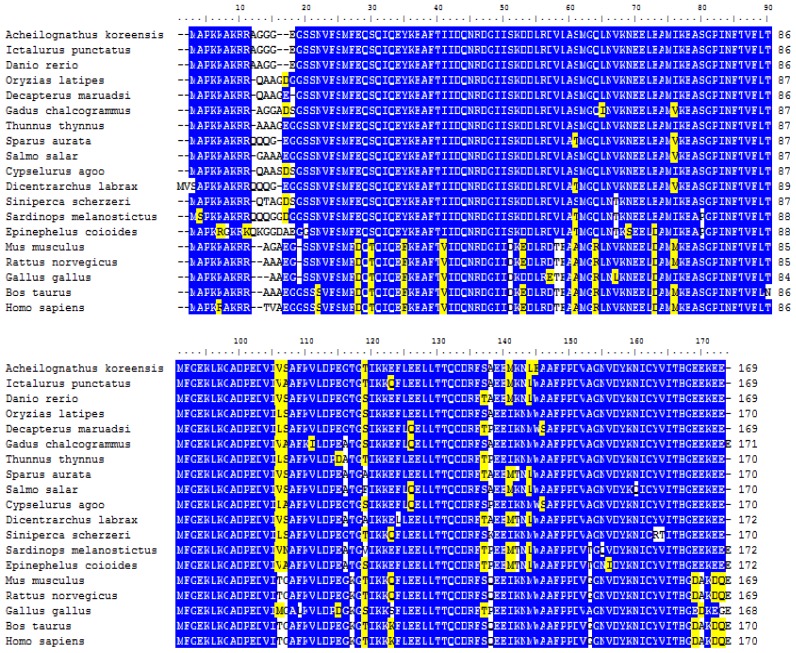
Multiple alignment of AkMLC2f amino acid sequences with MLC2 from other species. Identical residues in all sequences are indicated in blue. Conserved substitutions are indicated in yellow. The sequences were extracted from GenBank: *Ictalurus punctafus* (NP_001188138), *Danio rerio* (NP_571263), *Sardinops melanostictus* (BAA95140), *Epinephelus coioides* (ACM41847), *Sparus aurata* (AAD54229), *Dicentrarchus labrax* (CBN81401), *Salmo salar* (NP_001117188), *Gadus chalcogrammus* (BAB18578), *Oryzias latipes* (XP_004071230), *Decapterus maruadsi* (BAB69803), *Cypselurus agoo* (BAA95134), *Thunnus thynnus* (BAA95125), *Siniperca scherzeri* (ACT67908), *Gallus gallus* (NP_001185673), *Mus musculus* (NP_058034), *Rattus norvegicus* (NP_036737), *Bos Taurus* (NP_001069115), and *Homo sapiens* (AAA91848).

Using the BioEdit software, pairwise alignment revealed amino acid identities of 80%–98% in MLC2f of *A. koreensis* and other species, including fish, birds and mammals (Figure 3). Multiple alignment revealed that MLC2 shared the highly conserved EF hand motif and putative phosphorylation site at the *N*-terminal serine residue [1]. In the Ca^2+^ binding domain, fish shared the Ser residue while the remainder shared an Asp residue. These results suggest that AkMLC2f is evolutionarily highly conserved.

### 2.3. Promoter Activity of *Akmlc2f*

**Figure 4:**
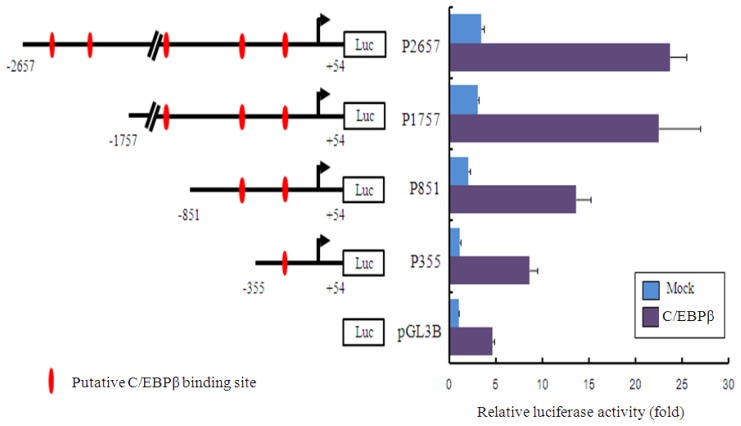
Analysis of luciferase reporter activity of the *Akmlc2f* promoter. Cells were seeded in 24-well culture plates and transfected with the indicated reporter vectors, (pcDNA3/C/EBPβ and β-galactosidase expression plasmids). At 24 h after transfection, the cells were lysed and assayed for the luciferase reporter activity. The data are representative of two independent experiments. The values represent means ± SD (*n* = 3).

**Figure 5:**
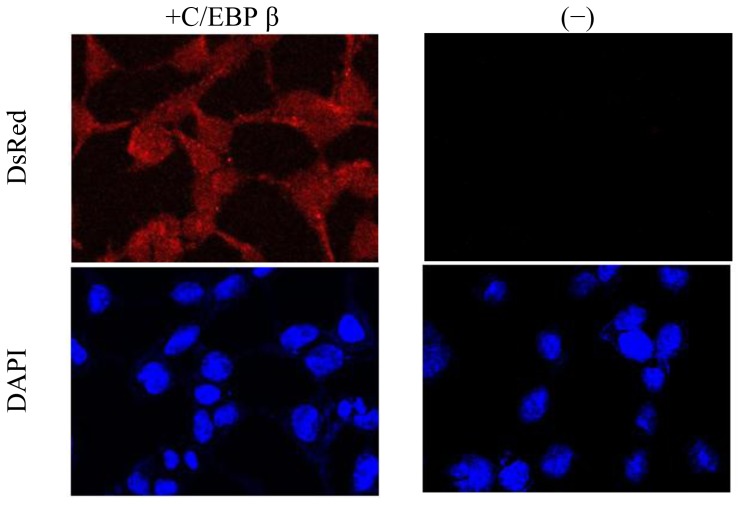
Analysis of DsRed reporter activity of the *Akmlc2f* promoter. Cells were seeded in 12-well culture plates and transfected with a reporter vector (pDsRed2-1/Akmlc2f P2657) and pcDNA3/C/EBPβ or empty expression plasmid. At 24 h after transfection, the cells were fixed with 4% paraformaldehyde in PBS (pH 7.4), mounted using VECTASHIELD^®^ with DAPI, and viewed using a confocal laser scanning microscope.

**Figure 6:**
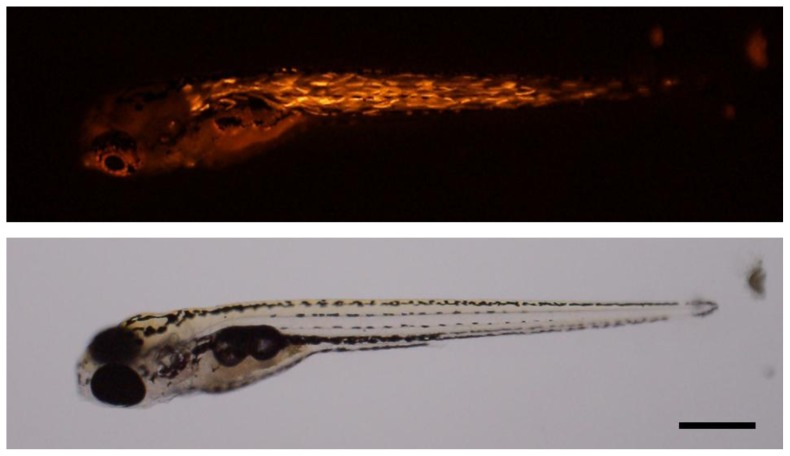
Analysis of DsRed reporter activity of the *Akmlc2f* promoter in the zebrafish embryo. The fluorescent protein reporter of the *Akmlc2f* promoter, pDsRed2-1/Akmlc2f P2657 was digested with the restriction endonuclease *Apa*LI and then the DNA solution at concentrations of 25 ng/mL was injected into the one-cell stage embryos using an air-pressure microinjector (WPI). Digital images of embryo (6 days post hatching) were captured using a macro zoom fluorescence microscope (Olympus, Tokyo, Japan). Scale bar = 200 μm

MEF2 involved in the regulation of muscle development can regulate gene expression by binding the promoter of teleost *mlc* genes [18]. The GATA motif and serum response factor (SRF) site within the promoter region of the frog *mlc* gene act synergistically to regulate the *mlc2* gene [19,20]. We identified the potential transcriptional binding sites using the TFSEARCH software (http://www.cbrc.jp/research/db/TFSEARCH.html, AIST, Tokyo, Japan). A putative TATA box (TATATAA) and one copy of a putative MEF-2 binding site were found at (−30)~(−24) bp from the putative transcription start site and in the proximal promoter region, respectively (data not shown). Among the various transcription-factor-binding sites, several putative CCAAT-enhancer-binding protein beta (C/EBPβ)-binding sites were identified as indicated in Figure 4. C/EBPβ is a ZIP transcription factor that can bind as a homodimer or heterodimer with C/EBPα and C/EBPγ to certain promoters and enhancers [21]. C/EBPβ is important for the regulation of genes involved in immune and inflammatory responses, and increased C/EBPβ expression in blood leukocytes is positively associated with greater muscle strength in humans [22]. Lyons and co-authors identified c/ebpα, β, γ, δ in zebrafish and suggested that the expression of c/ebps genes serve as analytical markers for developmental processes including myelopoiesis and hepatic development [23]. We performed a promoter assay to assess the *Akmlc2f* gene with C/EBPβ in HEK293T cells. Compared to the luciferase activity in cells transfected with empty pGL3B vector and C/EBPβ, the activity of the constructs (pGL3B, P355, P851, P1757, and P2657) was 4.5-, 7.8-, 6.5-, 7.4-, and 6.9-fold higher, respectively. The results suggest that the first putative C/EBPβ binding element at (−87)~(−74) bp from the transcription initiation site plays a role in the transactivation of the *Akmlc2f* gene by C/EBPβ. Vertebrate *mlc2* promoters for efficient muscle-specific expression can be relatively short; for example, the *mlc2* promoter is 64 bp in chicken and 79 bp in zebrafish [10,11,24]. To visualize the activation of the *Akmlc2f* promoter, we performed a fluorescence microscopic analysis with the *Akmlc-2* promoter (P2657)-driven DsRed2 gene construct in HEK293T cells. The *Akmlc2f* promoter (P2657)-driven DsRed2 expression by C/EBPβ was detected in the cytoplasm and nucleus (Figure 5). In addition, we performed a fluorescence microscopic analysis with the *Akmlc2f* promoter (P2657)-driven DsRed2 gene construct in the developing zebrafish embryo. The red fluorescence signal was mainly observed in muscular area of the developing zebrafish embryo at 6 days post hatching (Figure 6). This is in agreement with the expression pattern of *mlc2f* promoter-driven fluorescent reporters in marine medaka and Javanese Ricefish [13,14].

### 2.4. Expression of *Akmlc2f* mRNA

**Figure 7:**
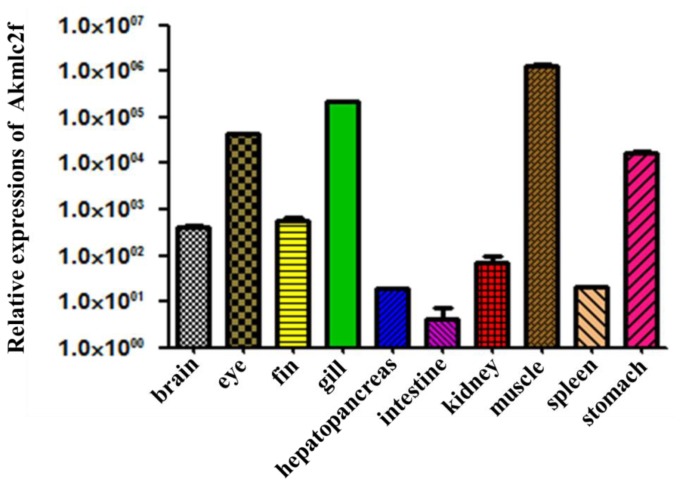
Tissue distribution of *Akmlc2f* mRNA. Quantitative real-time polymerase chain reaction was performed on equal amounts of total RNA isolated from the *A. koreensis* tissues. To determine tissue-specific expression levels, the expression level in each tissue was compared to that in the intestine. Values are means ± SD (*n* = 3).

To examine the tissue distribution of *Akmlc2f* mRNA, quantitative real-time polymerase chain reaction (PCR) was performed on various *A. koreensis* tissues (Figure 7). *Akmlc2f* mRNA was highly enriched in skeletal muscles and was detectable in other tissues, albeit weakly. Muscle exhibited the highest expression level; however, *Akmlc2f* expression was relatively high in the eye and gill. The lowest level of *Akmlc2f* mRNA was found in intestine (smooth muscle), which was similar to the level in hepatopancreas. The expression patterns of the *Akmlc2f* transcript are similar to those of marine medaka *mlc2f* transcripts [13]. In addition to Korean oily bitterling and marine medaka, skeletal muscle-enriched expression of *mlc2f* has been reported in gilthead sea bream and zebrafish [6,10,25].

## 3. Materials and Methods

### 3.1. Isolation and Sequencing of *A. koreensis Akmlc2f* cDNA

The EST clones were isolated from an *A. koreensis* cDNA library using a plasmid miniprep kit (Qiagen, Seoul, Korea), and sequenced using T3 reverse primers (Promega, Madison, WI, USA) on an ABI3730xl automatic sequencer (Applied Biosystems, Inc., Foster City, CA, USA). The EST clone AK-1-2a-O18, which carries a 1247-bp insertion, showed significant sequence homology to the known MLC-2 sequences. The 5′-UTR of *Akmlc2f* was amplified from the cDNA of muscle tissue using 5′-RACE using the SMART RACE cDNA amplification kit (Clontech, Mountain View, CA, USA). Based on a partial sequence, we designed internal primers (*Akmlc2f*-5R2, 5′-CTC AAG GAA TTC CTT CTT GAT GGT G-3′) and used them in combination with the universal primer supplied with the kit to amplify the 5′-end of the *Akmlc2f* transcript. Analyses of a potential ORF and comparisons of the *Akmlc2f* amino acid sequence (or nucleotide sequence) were performed using the ORF finder and BLAST programs (http://www.ncbi.nlm.nih.gov/BLAST/, NCBI, Bethesda, MD, USA). The theoretical pI and MW of the deduced AkMLC2f were computed on the ExPASy website (http://web.expasy.org/compute_pi/, SIB, Lausanne, Switzerland).

### 3.2. Isolation and Sequence Analysis of *A. koreensis mlc2f* Gene

An *A. koreensis* fosmid library was screened to isolate the *Akmlc2f* gene using the fosmid pooling system with PCR primers specific for the *Akmlc2f* cDNA. PCR-based fosmid library screening was carried out as described previously [26]. The obtained *Akmlc2f* genomic clone #17-H18 was purified and used to determine the nucleotide sequence and genomic structure.

### 3.3. Sequence Alignments

Pair-wise and multiple sequence alignments were performed using BioEdit software (http://www.mbio.ncsu.edu/bioedit/bioedit.html, Ibis Biosciences, Carlsbad, CA, USA). The sequences were extracted from GenBank: *Ictalurus punctafus* (NP_001188138), *Danio rerio* (NP_571263), *Sardinops melanostictus* (BAA95140), *Epinephelus coioides* (ACM41847), *Sparus aurata* (AAD54229), *Dicentrarchus labrax* (CBN81401), *Salmo salar* (NP_001117188), *Gadus chalcogrammus* (BAB18578), *Oryzias latipes* (XP_004071230), *Decapterus maruadsi* (BAB69803), *Cypselurus agoo* (BAA95134), *Thunnus thynnus* (BAA95125), *Siniperca scherzeri* (ACT67908), *Gallus gallus* (NP_001185673), *Mus musculus* (NP_058034), *Rattus norvegicus* (NP_036737), *Bos Taurus* (NP_001069115), and *Homo sapiens* (AAA91848).

### 3.4. Construction of Reporter Plasmids

To examine the functional activity of the *Akmlc2f* promoter, the DNA fragments, ranging from positions −2657 to +54 bp in the *Akmlc2f* gene, were generated by PCR using Vent DNA polymerase (New England BioLabs, Beverly, MA, USA). The primers were designed so that the amplified DNA would contain *Mlu*I and *Xho*I restriction sites at its 5′ and 3′ ends, respectively. The primer sequences were: *Akmlc2f*-2657FM, 5′-GCG ACG CGT CAG TTT TTA GTT TAT TTT AC-3′; *Akmlc2f*-1757FM, 5′-GTC ACG CGT CAT GTA CTG TAT GTA TAC AT-3′; *Akmlc2f*-851FM, 5′-GTT ACG CGT AGA AAG GAG GAT AAT TAC GT-3′ *Akmlc2f*-355FM, 5′-GCT ACG CGT ATT GAT CCA CCA CTA ATC CA-3′ and *Akmlc2f*-+54RX, 5′-GTT CTC GAG CAT GCT GGG ACG GTG TGA AG 3′. The amplified cDNA fragments were inserted into the *Mlu*I and *Xho*I restriction sites upstream of a luciferase gene in the pGL3-Basic vector (Promega, Madison, WI, USA). To create the fluorescent protein reporter of the *Akmlc2f* promoter, the DNA fragments ranging from positions −2657 to +54 bp in the *Akmlc2f* gene, were generated by PCR using Vent DNA polymerase (New England BioLabs, Beverly, MA, USA) and then inserted into the *Sal*I and *Age*I restriction sites upstream of a *DsRed2* gene in a vector (pDsRed2-1, Clontech, Mountain View, CA, USA). The primer sequences were as follows: *Akmlc2f*-2657FS, 5′-CGA GTC GAC CAG TTT TTA GTT TAT TTT AC-3′ and *Akmlc2f*-+54RA, 5′-GAT ACC GGT CAT GCT GGG ACG GTG TGA AG 3′. The constructs were confirmed by sequencing.

### 3.5. Cell Culture, Transient Transfection and Luciferase Assay

HEK293T cells were maintained in Dulbecco’s Modified Eagle’s medium (Gibco–BRL, Gaithersburg, MD, USA) with 10% heat-inactivated fetal bovine serum (FBS; Gibco–BRL, Gaithersburg, MD, USA) and 1% (*v*/*v*) penicillin-streptomycin (PS; Gibco–BRL, Gaithersburg, MD, USA) in a 37 °C incubator. For the luciferase assay, cells were seeded in 24-well culture plates and transfected with each indicated reporter vector (pcDNA3/C/EBPβ and β-galactosidase expression plasmid) using Polyfect transfection reagent (Qiagen, Seoul, Korea). After 24 h of transfection, the cells were lysed in cell culture lysis buffer (Promega, Madison, WI, USA). Luciferase activity was determined using an analytical luminometer (Wallac Victor2 plate reader, Perkin Elmer, Waltham, MA, USA), according to the manufacturer’s instructions (Promega, Madison, WI, USA). The luciferase activity was normalized for transfection efficiency using the corresponding β-galactosidase activity. All assays were performed in at least triplicate. All data are reported as means ± SD.

### 3.6. Confocal Microscopy

HEK293T cells were seeded on glass coverslips in a 12-well plate and transiently transfected with 400 ng of pcDNA3/C/EBPβ or empty vector with 400 ng of pDsRed2-1/Akmlc2f P2657 using Polyfect transfection reagent (Qiagen, Seoul, Korea). At 24 h after transfection, the cells were fixed with 4% paraformaldehyde in PBS (pH 7.4), mounted using VECTASHIELD^®^ with DAPI (Vector Labs, Burlingame, CA, USA), and viewed using an LSM700 confocal laser scanning microscope (Carl Zeiss, Oberkochen, Germany). The promoter activity of pDsRed2-1/Akmlc2f P2657 with C/EBPβ or empty vector in the cells was demonstrated by direct fluorescence; stacks of optical sections were acquired by sequential acquisition and analyzed using the LSM software (ZEN 2009, Carl Zeiss, Oberkochen, Germany).

### 3.7. Microinjection into Zebrafish Embryo

Adult fish were maintained at 28.5 °C with a 14 h light/10 h dark cycle as described in Westerfield [27]. The fluorescent protein reporter of the *Akmlc2f* promoter, pDsRed2-1/Akmlc2f P2657 was digested with the restriction endonuclease *Apa*LI (New England BioLabs, Beverly, MA, USA). The DNA solution at concentrations of 25 μg/mL was injected into the one-cell stage embryos using an air-pressure microinjector (PicoPump, WPI, Sarasota, FL, USA) as described previously [28]. The estimated amount of DNA delivered to each embryo was 20 pg on average. Digital images of embryos were captured using a macro zoom fluorescence microscope (MVX10, Olympus, Tokyo, Japan).

### 3.8. Fish and Tissue Samples

*A. koreensis* specimens were collected from the Deokchun River, Sangcheong-gun, Gyungnam, Korea. The fish were maintained at the National Fisheries Research and Development Institute (NFRDI) in Busan, Korea. The adults were maintained in 40-L glass aquaria at a density of approximately 20 fish per aquarium. The water was renewed every other day and the temperature in the rearing tanks was maintained at 18 ± 1 °C. The room was maintained on a 12:12-h light-dark cycle. Adults were fed TetraBits (Tetra, Melle, Germany) and frozen bloodworms (Advanced Hatchery Technology, Salt Lake City, UT, USA) twice a day. For RNA extraction, tissues were removed from three fish, immediately frozen in liquid nitrogen, and stored at −80 °C before use.

### 3.9. Quantitative Real-Time PCR

Total RNA was prepared from tissues using TRIzol reagent (Invitrogen, Carlsbad, CA, USA) according to the manufacturer’s instructions, treated with DNase I (New England BioLabs, Beverly, MA, USA) and quantitatively determined; 1-μg samples were used for reverse transcription (RT). First-strand cDNA was synthesized using an Advantage RT-for-PCR kit (BD Sciences, San Jose, CA, USA). Quantitative real-time PCR was performed using Fast SYBR Green Master Mix (Applied Biosystems, Inc., Foster City, CA, USA) and the following forward and reverse primers: *Akmlc2f*, *Akmlc2f*-RT-F (5′-GCT TCA GTC TTC ACT TCT TGA GCT T-3′) and *Akmlc2f*-RT-R (5′-CCT CCT GCC CTC CTC TTA GC-3′). Following an initial 10-min Taq activation step at 95 °C, real-time PCR was performed for 40 cycles using the following conditions: 95 °C for 10 s, 60 °C for 15 s. Fluorescence readings were performed using SDS 7500 (Applied Biosystems, Inc., Foster City, CA, USA), and analyzed by the melt curve to check the specificity of the PCR product.

### 3.10. Statistical Analysis

All data are expressed as means ± SD (*n* = 3). Statistical significance was determined using the unpaired two-tailed Student’s *t*-test. *p* values less than 0.05 were considered to represent statistically significant differences.

## 4. Conclusions

In this study, we report the molecular cloning and characterization of the *mlc2f* gene of Korean oily bitterling *Acheilognathus koreensis* at both the mRNA and genomic DNA levels. We report that the genomic organization of *Akmlc2f* includes a seven-exon structure, which is conserved in vertebrate orthologs, and short duplicated sequences in intron 1. In addition, we showed that high levels of *Akmlc2f* transcripts are expressed in skeletal muscle but not in smooth muscle. Moreover, we showed that the activities of the *Akmlc2f* promoter might be regulated by the C/EBPβ transcription factor in the luciferase or DsRed reporter assay system using deletion constructs (P355, P851, P1757 and P2657). The activity of the promoter region of *Akmlc2f* was also confirmed in the developing zebrafish embryo. Taken together, these results lay the groundwork for investigating factors that regulate *Akmlc2f* expression and for developing a useful promoter for gene expression in fish.

## References

[1] England J., Loughna S. (2013). Heavy and light roles: Myosin in the morphogenesis of the heart. Cell Mol. Life Sci.

[2] Harrington W.F., Rodgers M.E. (1984). Myosin. Annu. Rev. Biochem.

[3] Cheney R.E., Riley M.A., Mooseker M.S. (1993). Phylogenetic analysis of the myosin superfamily. Cell Motil. Cytoskeleton.

[4] Warrick H.M., Spudich J.A. (1987). Myosin structure and function in cell motility. Annu. Rev. Cell Biol.

[5] Czosnek H., Nudel U., Shani M., Barker P.E., Pravtcheva D.D., Ruddle F.H., Yaffe D. (1982). The genes coding for the muscle contractile proteins, myosin heavy chain, myosin light chain 2, and skeletal muscle actin are located on three different mouse chromosomes. EMBO J.

[6] Moutou K.A., Canario A.V., Mamuris Z., Power D.M. (2001). Molecular cloning and sequence of Sparus aurata skeletal myosin light chains expressed in white muscle: Developmental expression and thyroid regulation. J. Exp. Biol.

[7] Ikebe M., Hartshorne D.J. (1985). Phosphorylation of smooth muscle myosin at two distinct sites by myosin light chain kinase. J. Biol. Chem.

[8] Venema R.C., Raynor R.L., Noland T.A., Kuo J.F. (1993). Role of protein kinase C in the phosphorylation of cardiac myosin light chain 2. Biochem. J..

[9] Wilkinson S., Paterson H.F., Marshall C.J. (2005). Cdc42-MRCK and Rho-ROCK signalling cooperate in myosin phosphorylation and cell invasion. Nat. Cell Biol.

[10] Xu Y., He J., Tian H.L., Chan C.H., Liao J., Yan T., Lam T.J., Gong Z. (1999). Fast skeletal muscle-specific expression of a zebrafish myosin light chain 2 gene and characterization of its promoter by direct injection into skeletal muscle. DNA Cell Biol.

[11] Krasnov A., Teerijoki H., Gorodilov Y., Molsa H. (2003). Cloning of rainbow trout (*Oncorhynchus mykiss*) α-actin, myosin regulatory light chain genes and the 5′-flanking region of α-tropomyosin. Functional assessment of promoters. J. Exp. Biol.

[12] Funkenstein B., Skopal T., Rapoport B., Rebhan Y., Du S.J., Radaelli G. (2007). Characterization and functional analysis of the 5′ flanking region of myosin light chain-2 gene expressed in white muscle of the gilthead sea bream (*Sparus aurata*). Comp. Biochem. Physiol. Part D Genomics Proteomics.

[13] Cho Y.S., Lee S.Y., Kim D.S., Nam Y.K. (2013). Characterization of stable fluorescent transgenic marine medaka (*Oryzias dancena*) lines carrying red fluorescent protein gene driven by myosin light chain 2 promoter. Transgenic Res.

[14] Lee S.Y., Kim D.S., Nam Y.K. (2012). Genomic organization, intronic duplications, and promoter characteristics of the fast skeletal myosin light chain-2 gene (*mlc2f*) from javanese ricefish *Oryzias javanicus*. Fish Aquat. Sci.

[15] Kim I.S., Choi Y., Lee C.L., Lee Y.J., Kim B.Y., Kim J.H. (2005). Illustrated Book of Korean Fishes (in Korean).

[16] Hwang D.S., Lee W.O., Lee J.S. (2013). Complete mitochondrial genome of the Korean bitterling *Acheilognathus koreensis*(Cypriniformes; Cyprinidae). Mitochondrial DNA.

[17] Ogut O., Brozovich F.V. (2003). Regulation of force in vascular smooth muscle. J. Mol. Cell Cardiol.

[18] Steinbacher P., Haslett J.R., Six M., Gollmann H.P., Sänger A.M., Stoiber W. (2006). Phases of myogenic cell activation and possible role of dermomyotome cells in teleost muscle formation. Dev. Dynam.

[19] Latinkic B.V., Cooper B., Smith S., Kotecha S., Towers N., Sparrow D., Mohun T.J. (2004). Transcriptional regulation of the cardiac specific *Mlc2* gene during *Xenopus* embryonic development. Development.

[20] Gove C., Walmsley M., Nijjar S., Bertwistle D., Guille M., Partington G., Bomford A., Patient R. (1997). Over-expression of GATA-6 in *Xenopus* embryos blocks differentiation of heartprecursors. EMBO J.

[21] Ramji D.P., Foka P. (2002). CCAAT/enhancer-binding proteins: Structure, function and regulation. Biochem. J.

[22] Harries L.W., Pilling L.C., Hernandez L.D., Bradley-Smith R., Henley W., Singleton A.B., Guralnik J.M., Bandinelli S., Ferrucci L., Melzer D. (2012). CCAAT-enhancer-binding protein-beta expression *in vivo* is associated with muscle strength. Aging Cell.

[23] Lyons S.E., Shue B.C., Lei L., Oates A.C., Zon L.I., Liu P.P. (2001). Molecular cloning, genetic mapping, and expression analysis of four zebrafish *c/ebp* genes. Gene.

[24] Braun T., Tannich E., Buschhausen-Denker G., Arnold H.H. (1989). Promoter upstream elements of the chicken cardiac myosin light-chain 2-A gene interact with trans-actin regulatory factors for muscle-specific transcription. Mol. Cell. Biol.

[25] Ju B., Chong S.W., He J., Wang X., Xu Y., Wan H., Tong Y., Yan T., Korzh V., Gong Z. (2003). Recapitulation of fast skeletal muscle development in zebrafish by transgenic expression of GFP under the mylz2 promoter. Dev. Dyn.

[26] Chae S.H., Kim J.W., Cho J.M., Larkin D.M., Everts-van der Wind A., Park H.S., Yeo J.S., Choi I. (2007). Chromosomal localization of *Korean cattle* (Hanwoo) BAC clones via BAC end sequence analysis. Asian Australas. J. Anim. Sci.

[27] Westfield M. The Zebrafish Book.

[28] Kikuchi Y., Segawa H., Tokumoto M., Tsubokawa T., Hotta Y., Uyemura K., Okamoto H. (1997). Ocular and cerebellar defects in zebrafish induced by overexpression of the LIM domains of the islet-3 LIM/homeodomain protein. Neuron.

